# Molecular Nanoinformatics Approach Assessing the Coating Oxcarbazepine (OXC) Drug on Silver Nanoparticles

**DOI:** 10.1021/acsomega.4c06366

**Published:** 2024-11-05

**Authors:** Norberto de Kássio Vieira Monteiro, Lucas Lima Bezerra, Leonardo P. da Silva, Richele Machado

**Affiliations:** † Department of Analytical Chemistry and Physical Chemistry, Science Center, Federal University of Ceará, 60020-181 Fortaleza, CE, Brazil; ‡ Christus University Center, 60160-230 Fortaleza, CE, Brazil

## Abstract

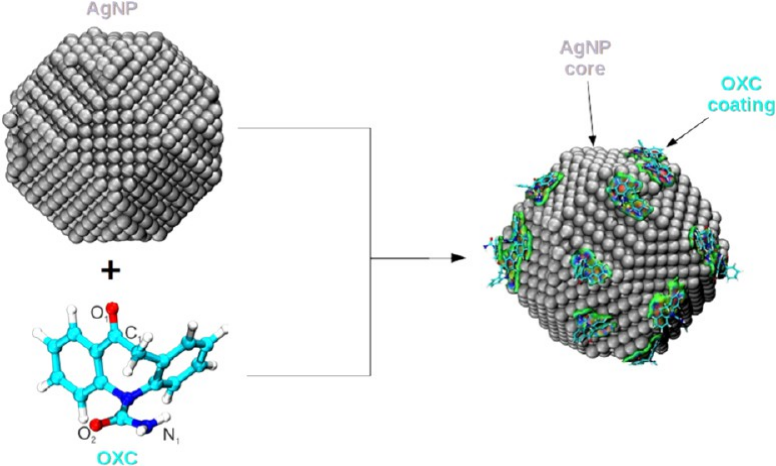

Silver nanoparticles (AgNP) have gained significant attention due to their unique pharmacological properties. These nanoparticles have been found to possess antimicrobial, anti-inflammatory, and antioxidant activities, making them promising candidates for various medical applications. The coating characteristics of oxcarbazepine (OXC), a drug used in epilepsy treatment, on the AgNP icosahedral clusters were investigated using molecular dynamics (MD) simulations and noncovalent interactions (NCI) and Independent Gradient Model (IGM) analysis. We investigated the AgNP coating using OXC drug concentrations of 500, 1000, 1500, 2000, and 2500 ppm. Our results suggested that the OXC drug has a high potential interaction with the AgNP, especially when the concentration increases. Furthermore, it was observed that this interaction occurs mainly through the nitrogen atom (N_1_) of the OXC molecule, independent of concentration. Finally, the coating is more pronounced for high OXC concentrations. The weak interaction analysis indicated that the van der Waals interactions were observed between the OXC molecules and AgNP, resulting in relevant stability in these interactions. Therefore, our study may be helpful for experimental research groups to develop an oxcarbazepine drug delivery system using AgNP.

## 1 Introduction

Anticonvulsants are a crucial component in the treatment of neurological disorders caused by seizures, as well as epilepsy.^1^ Anticonvulsants work through various mechanisms, including modulation of γ-aminobutyric acid (GABA) and glutamate neurotransmission, ion channel modulation, and alteration of synaptic transmission.^2−4^ Additionally, anticonvulsants have been shown to reduce cortical excitability in epilepsy, indicating their efficacy in managing the condition.^5^ However, the use of anticonvulsants is not without adverse effects. Some anticonvulsants have been associated with disruptions in sleep patterns, leading to increased drowsiness in patients.^6^ Furthermore, the tolerability of newer anticonvulsants has been a subject of study, with some preliminary findings suggesting that they may be less well-tolerated than older drugs.^7^ Additionally, the management of anticonvulsants in children with epilepsy is crucial to minimize breakthrough seizures and associated morbidity and mortality.^8,9^

Oxcarbazepine (10,11-dihydro-10-oxo-5*H*-dibenzo-[*b*,*f*]azepine-5-carboxamide, OXC) is an antiepileptic drug for managing epilepsy. It is effective in treating partial-onset seizures in both adults and children.^10^ The OXC shares a common primary mechanism of action with carbamazepine, which blocks high-frequency repetitive firing of sodium channels.^11^ Its efficacy and tolerability have been observed in adult patients with newly diagnosed or refractory partial epilepsy.^12^ However, some studies have suggested its ineffectiveness in controlling seizures, particularly in certain types of epilepsy.^13,14^

Besides, the OXC has a bioavailability of more than 95%, a maximum concentration of around 1–3 h, and a life of 1 to 5 h.^15,16^ The metabolization occurs by the aldo keto reductase, resulting in a hydroxylation, deriving in the 10,11-dihydro-10-hydroxy-5*H*-dibenz[*b*,*f*]azepine-5-carboxamide (MHD), this being the main agent in antiepileptic action having similar efficacy to OXC.^17^ Furthermore, Flesch et al.^18^ showed that the aliment ingestion did not significantly change the OXC absorption, with the recommended dose between 600 and 2400 mg/day.^19^ Moreover, its action is dose-dependent, and oral administration of OXC may lead to viability to other routes and organs of no interest, which can increase the risk of unfavorable dose-dependent side effects. A study on interindividual variability indicates that monitoring MHD plasma concentrations is interesting.^20^ Therefore, the use of the drug with a controlled release may be welcomed to manage its side effects better.

Researchers have explored alternative delivery methods to improve the drug’s efficacy in response to this ineffectiveness and, for instance, formulated oxcarbazepine-loaded PLGA nanoparticles for intranasal delivery, demonstrating effective control of epileptic seizures in rodents.^21^ Additionally, a study successfully utilized magnetic nanoparticles to extract oxcarbazepine from biological samples, showcasing the potential for improved drug monitoring and personalized treatment.^22^ Furthermore, a survey highlighted the enhanced distribution of oxcarbazepine in the brain through intranasal administration of PLGA nanoparticles.^23^

Among the nanoparticles, silver nanoparticles (AgNP) have been highlighted in recent years due to their unique electrical, optical, and catalytic properties. These unique properties have led researchers to employ the AgNP in the drug delivery, diagnosis, detection, and imaging area.^24,25^ However, the most interesting application of AgNP is associated with the biomedical area. This nanoparticle has high antibacterial,^26^ antifungal,^27^ and antimicrobial^28^ activities. Studies have shown that AgNP may induce oxidative stress and memory deficits in laboratory rats.^29^

In this paper, molecular dynamics (MD) simulations and aNCI and aIGM analyses were used to study the coating of five different concentrations of the OXC drug onto AgNP and to investigate the drug’s interaction potential on the surface of the AgNP.

## 2 Computational Details

### 2.1 OXC Optimization

The structure of the oxcarbazepine (OXC) was optimized by using the Gaussian 09 suite of programs. We use the hybrid functional B3LYP^30,31^ with a 6-311++G(d,p) basis set. Calculations of the vibrational frequencies of the optimized OXC geometry were performed to determine the nature of the stationary point. The optimization calculation was performed in solution by using the Polarizable Continuum Model (PCM)^32^ with the Integral Equation Formalism (IEF)^33^ using water as a solvent.

### 2.2 MD Simulations

The MD simulations were performed using GROMACS^34^ (Groningen MAchine for Chemical Simulation) *software* version 2023.2. This study’s silver nanoparticle (AgNP) comprised 2803 silver atoms. The AgNP in the icosahedral format was used as it is the most stable cluster for this system.^35^ The AgNP was built through the CHARMM-GUI nanomaterial modeler server.^36^ To investigate the effect of loading the OXC through the AgNP, we used five different concentrations of the OXC: 500, 1000, 1500, 2000, and 2500 ppm, which consisted of 17, 34, 51, 68, and 85 OXC molecules, respectively. The systems composed of AgNP and OXC molecules were placed in a simulation box and filled with water. The simulation box dimensions were 10 nm × 10 nm × 10 nm, and the distance between the AgNP coated and the box boundary was 2.7 nm. All simulations were conducted using the CHARMM36 force field^37^ under the periodic boundary conditions (PBCs) and the TIP3P model^38^ for water molecules. The Leap-Frog algorithm^39^ was applied to integrate the motion equation with a time step of 2.0 fs. The long-range interactions were modeled using particle-mesh Ewald sum (PME)^40^ with a cutoff of 1.2 nm. The van der Waals interactions were also calculated by using the same threshold. The bonds involving hydrogen atoms were restrained using the LINCS algorithm.^41^ The Nosé–Hoover thermostat^42^ was used to fix the system temperature at 310 K. The system pressure was controlled using a Parrinello–Rahman barostat^43^ at the value of 1.0 bar. The steepest descent^44^ and conjugate gradient^45^ algorithms were used to minimize the system’s energy for all atoms for 10^4^ steps with 10 kJ mol^–1^ nm^–1^ tolerance. Initially, each system was equilibrated through two steps: the first using an NVT ensemble for 125 ps followed by an NPT ensemble for 150 ps. Finally, each system was subjected to a 100 ns production MD simulation in three replicates through the NVT ensemble. The GROMACS utilities were used to analyze the trajectory data, and VMD^46^ 1.9.4 was used for molecular graphics and visualization.

**Figure 1:**
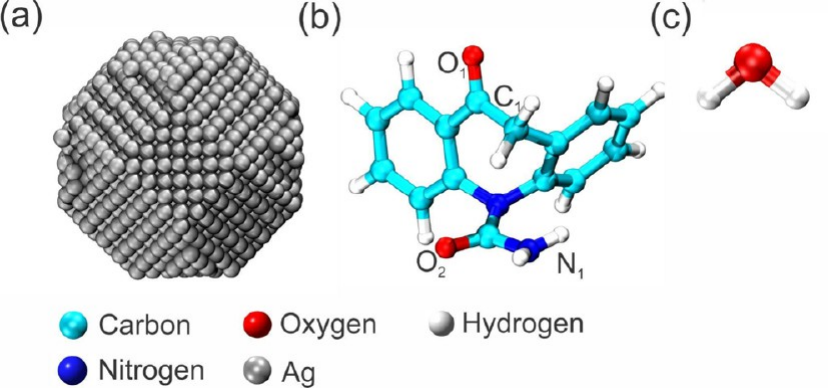
Tridimensional structures of (a) AgNP, (b) the OXC, and (c) the water species utilized in the MD simulations.

To analyze the interaction energy between OXC molecules and AgNP, we used the Interaction Potential Energy (IPE),^47^ which includes the sum of van der Waals and electrostatic contributions. Figure 1 shows the tridimensional structures of the species utilized in the MD simulations, with the nomenclature of the main atoms used in the radial distribution function (RDF) discussion.

### 2.3 Weak Interactions Analysis

The noncovalent interactions study (NCI) revealed the weak forces in systems OXC-AgNP. The NCI is based on the analysis of the reduced density gradient (RDG) (eq 1) as a function of the promolecular electron density (eq 2) and also as a function of the (sign λ_2_) ρ(*r*) representing the effective density, λ_2_ is the second-largest eigenvalue of the Hessian matrix of the electronic density (ρ).^48^ However, this work was analyzed by a dynamic system, so it is important to analyze it based on the trajectories of the molecule dynamics simulation. Therefore, the average value of the electron density will be considered, as well as the average value of the gradient of the electron density^49^ in eq 3.
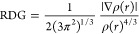
1
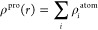
2
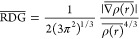
3

In addition, the OXC-AgNP interaction was analyzed by using the Independent Gradient Model (IGM). The function *g*(*r*) is the gradient of the electronic density between the atoms (eq 3). The *g*^IGM^ function is the type of gradient calculated by adding the absolute value of the density gradients of the atoms. The δ*g* function is the difference between *g* and *g*^IGM^, when δ*g* is different from 0 in the region between the nuclei. Still, in the interatomic region, it has a positive value, its maximum value being in the region of the critical bonding point.^50^
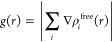
4
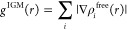
5
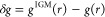
6

In the case of a fragment analysis, the region of interest is called fragment A for the nanoparticle, and its interaction region in the real space function,^50,51^ in a fragment in {A} is obtained from eqs 7–9.
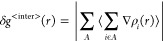
7
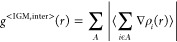
8
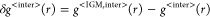
9where ρ_*i*_ is the promolecular electronic density of atom *i*, and averaged over all of the frames of the molecular dynamic trajectory, it is represented by <·>. In addition, the thermal fluctuation index (TFI) was calculated (eq 10), indicating the weak interaction’s stability.^49^
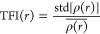
10
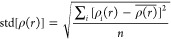
11

The term std[ρ(*r*)] is the standard deviation of electron density in the dynamical trajectory (eq 11), which can be calculated from eq 10, where *n* is the number of frames in the trajectory and ρ_*i*_(*r*) is the promolecular electron density of the geometry in frame *i*. The TFI can be analyzed by the difference in color, with blue regions being highly stable, green regions having medium interactions, and red regions indicating interactions that thermal movements can easily distort.^49^ All analyses of aNCI, aIGM, and TFI were performed using Multiwfn 3.8 software,^52,53^ and the images were rendered using Visual Molecular Studio software^46^ (VMD).

## 3 Results and Discussion

### 3.1 MD Results

**Figure 2:**
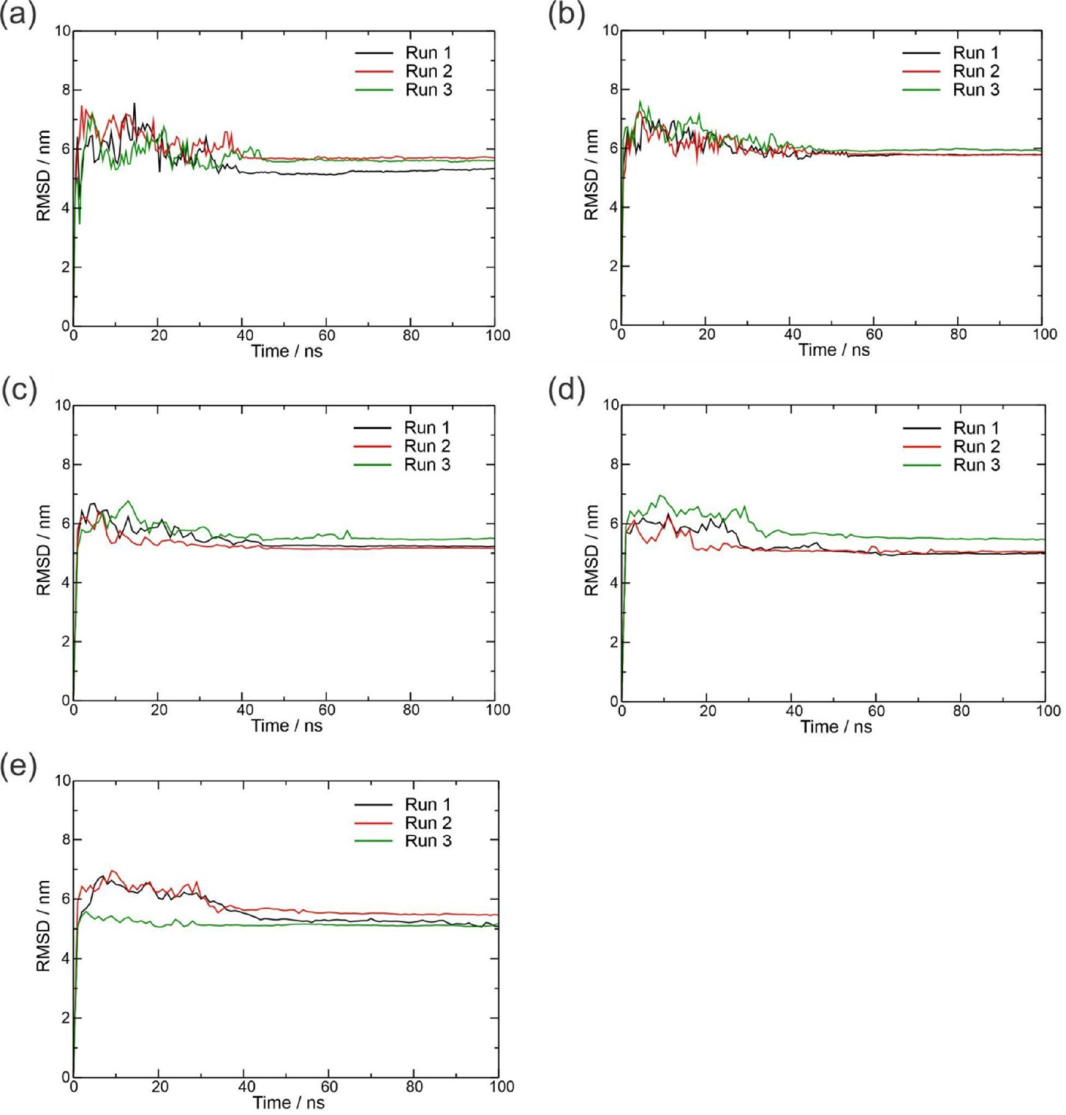
RMSD between the AgNP and OXC molecules with concentrations of (a) 500 ppm, (b) 1000, (c) 1500, (d) 2000, and (e) 2500 ppm. The black, red, and green lines represent each replicate simulated.

Figure 2 shows the root-mean-square deviation (RMSD) displacement between the OXC and the AgNP at five concentrations of the OXC. The black, red, and green lines represent each replicate simulated for these five systems. The systems simulated with the concentration OXC of 500 and 1000 ppm reached equilibrium around 45 ns, while the 1500, 2000, and 2500 ppm systems reached equilibrium around 40 ns. Therefore, the RDF analysis was based on the trajectories of MD simulations above.

**Figure 3:**
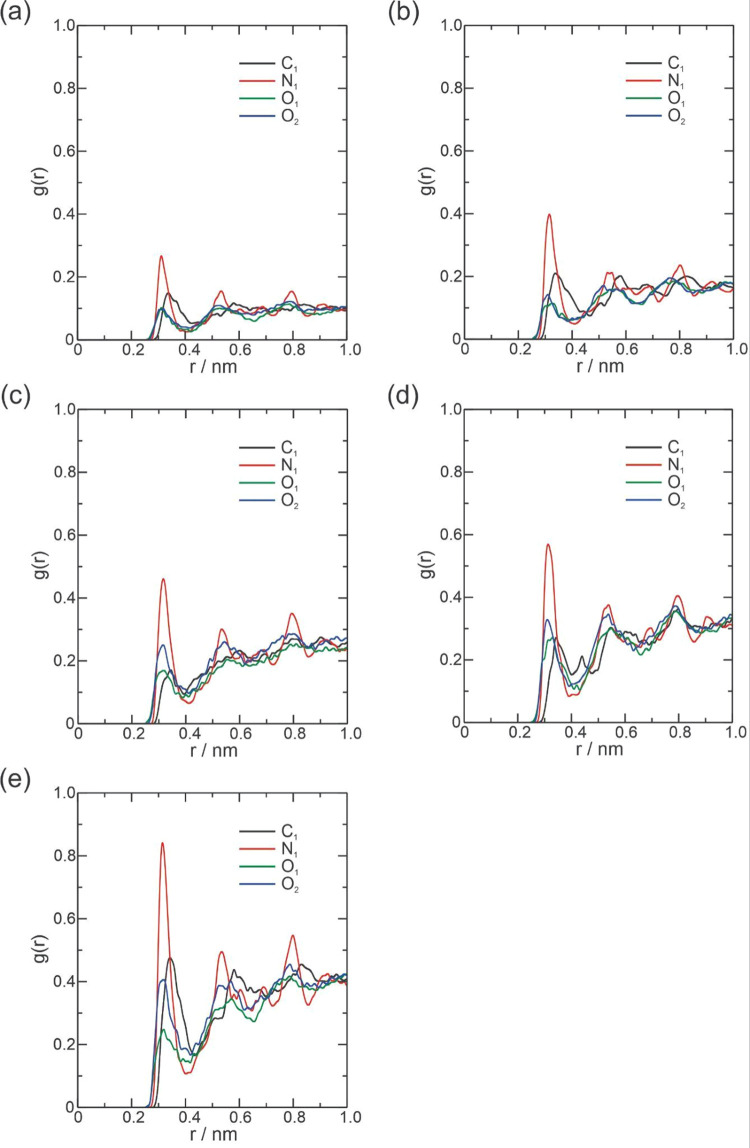
Radial distribution function plots for the specific regions of the OXC molecule (C_1_, N_1_, O_1_, and O_2_) in the concentrations of (a) 500 ppm, (b) 1000, (c) 1500, (d) 2000, and (e) 2500 ppm.

The radial distribution function (RDF, *g*(*r*) or pair correlation function) in a system describes how the density of molecules varies as a function of distance from a reference point.^54^ We investigated the arrangement of the OXC molecules and their affinity for interaction with AgNP through RDF analysis. Figure 3 depicts the RDF [*g*(*r*)] for specific regions of the OXC molecule at concentrations of 500 (Figure 3a), 1000 (Figure 3b), 1500 (Figure 3c), 2000 (Figure 3d), and 2500 ppm (Figure 3e) that interact with the AgNP.

For the system with 500 ppm (Figure 3a) of the OXC molecule, it was observed that the main interaction was between the nitrogen atom (N_1_) in the OXC molecule and the AgNP around 3.2 Å. The second strongest interaction registered was carbon atom C_1_ of the OXC with the AgNP around 3.4 Å. Furthermore, the oxygen atoms (O_1_ and O_2_) from the OXC molecule showed similar *g*(*r*) values with AgNP around 3.1 Å. Analyzing the effect of the increased OXC concentration, the same behavior in which interaction is more probable in the 500 ppm system is also observed for the other concentrations of OXC, however, with the highest *g*(*r*) values. The interaction between the nitrogen (N_1_), carbon (C_1_), and oxygen (O_2_) atoms with the AgNP registered the highest increase of *g*(*r*) values comparing the 500 ppm (Figure 3a) system with the 2500 ppm system (Figure 3e). Therefore, the RDF results suggested that the molecule of the OXC interacted mainly through nitrogen atoms (N_1_) with the AgNP. Furthermore, the probability increases when the concentration of the molecule of the AgNP is increased.

Table S1 shows the interaction potential energy (IPE) between the OXC molecules with AgNP at 500, 1000, 1500, 2000, and 2500 ppm concentrations in three replicates through the MD simulation. This calculation is obtained by the sum of short-range van der Waals and electrostatic energies. However, in this case, the IPE value is based only on the van der Waals energy due to the AgNP having charge zero; then, the electrostatic energy between the AgNP and the OXC molecules in all of the systems is zero. The IPE values reached the equilibrium around 50 and 60 ns for the 500 and 1000 ppm systems, respectively. On the other hand, the 1500 and 2000 ppm systems reached the equilibrium concerning the IPE values with the same interval of time (70 ns), while the 2500 ppm system reached around 80 ns. The average IPE values in equilibrium were −3410.68 kJ mol^–1^ (500 ppm), −6078.02 kJ mol^–1^ (1000 ppm), −8622.32 kJ mol^–1^ (1500 ppm), −10427.03 kJ mol^–1^ (2000 ppm), and −12100.80 kJ mol^–1^ (2500 ppm). Furthermore, the equilibrium IPE value for each replicate is present in Table S1 (Supporting Information). Therefore, the lowest IPE values between the OXC molecules and the AgNP in the 2500 ppm system were observed, indicating the highest interaction potential between these species, concerning the other systems.

**Figure 4:**
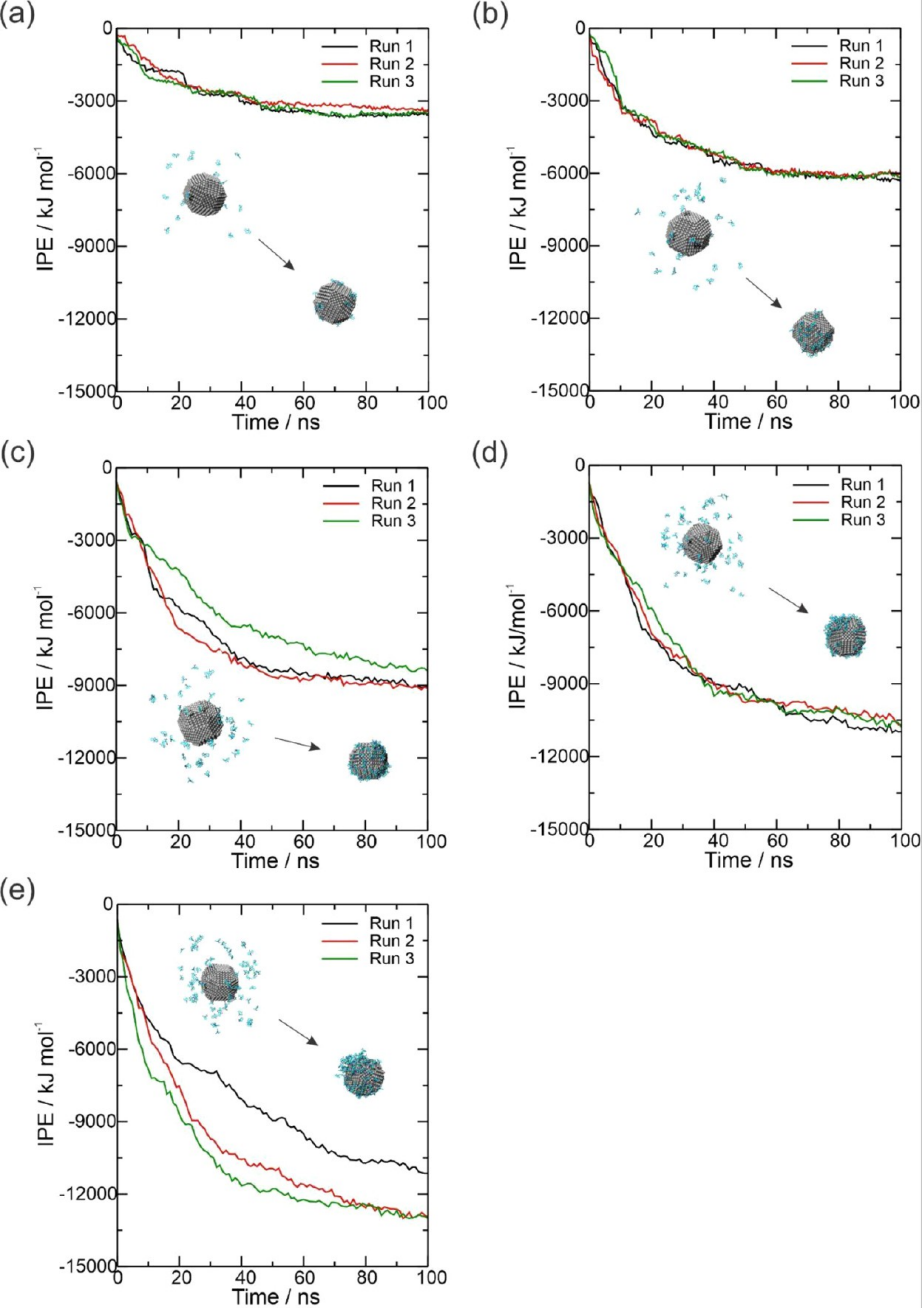
Plot of interaction potential energy (IPE), in kJ mol^–1^, as a function of time (in nanoseconds) obtained for the OXC molecules at concentrations of (a) 500 ppm, (b) 1000, (c) 1500, (d) 2000, and (e) 2500 ppm throughout the 100 ns MD simulation. The top inset depicts the initial structures of the AgNP surrounded by OXC molecules, and the bottom inset depicts the coated AgNP with OXC molecules after a 100 ns MD simulation.

The top inset of Figure 4a–e depicts the initial structures of the AgNP surrounded by the OXC molecules, while the bottom inset depicts the coated AgNP with the OXC molecules after a 100 ns MD simulation. We can see that at the end of the MD simulation (100 ns) for the five concentrations, the AgNP is coated with the OXC molecules. These findings are consistent with the above discussions of interaction affinity values obtained by IPE (see Figure 4), indicating that with increasing concentration of the OXC molecules, the affinity of the interaction with the OXC molecule increases, resulting in the highest coating by the OXC molecules.

**Figure 5:**
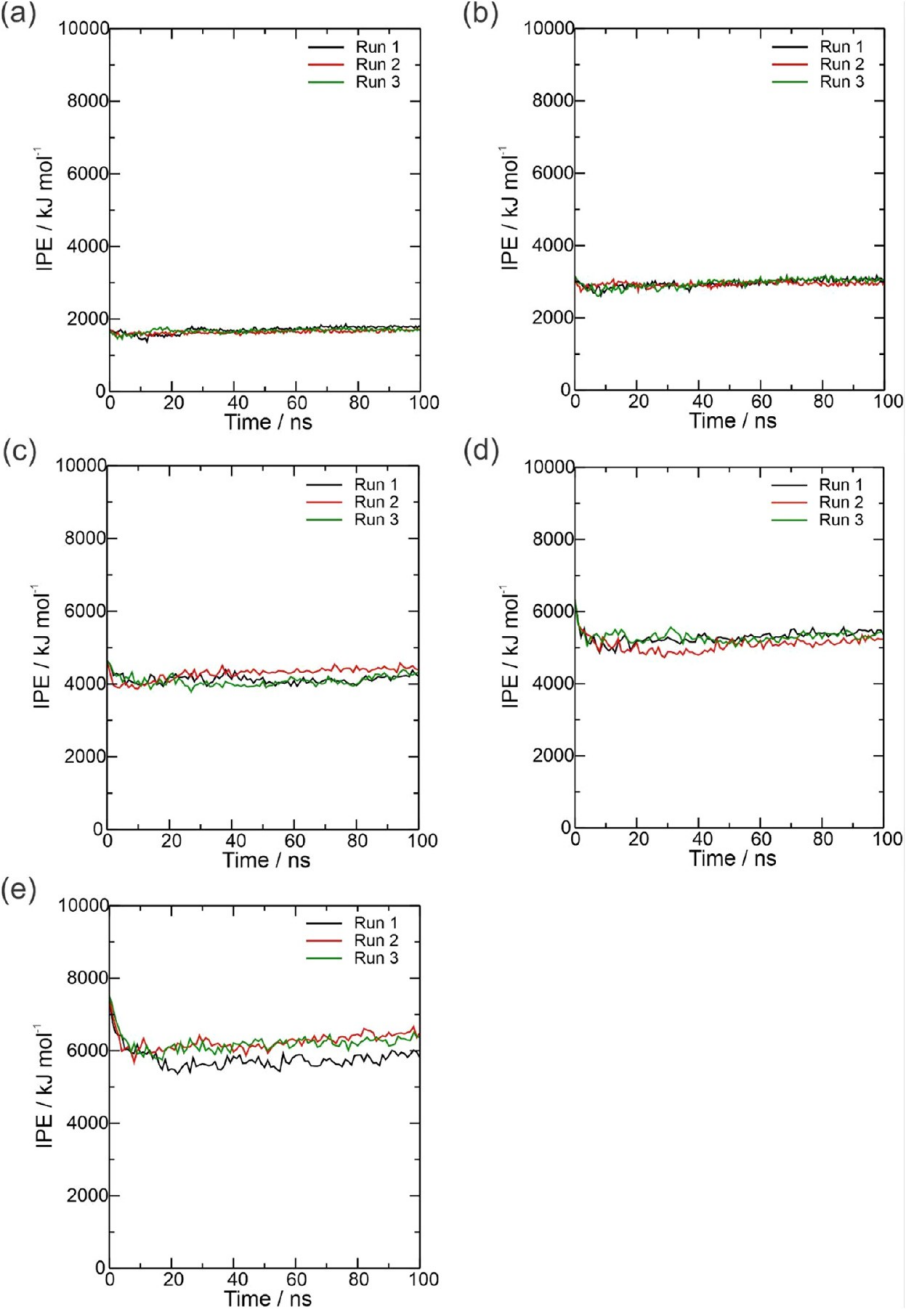
Plot of interaction potential energy (IPE), in kJ mol^–1^, as a function of time (in nanoseconds) obtained between the OXC molecules at concentrations of (a) 500 ppm, (b) 1000, (c) 1500, (d) 2000, and (e) 2500 ppm throughout the 100 ns MD simulation.

The IPE analysis was also utilized to understand the interactions between the OXC molecules in the five systems simulated (500, 1000, 1500, 2000, and 2500 ppm), as presented in Figure 5. The IPE values reached equilibrium at the beginning of simulations for the 500, 1000, 1500, and 2000 systems in Figure 5a–d, respectively. On the other hand, the IPE values reached equilibrium around 10 ns of simulations for the 2500 system (Figure 5e). The average IPE values in equilibrium were 1670.54 kJ mol^–1^ (500 ppm), 2948.87 kJ mol^–1^ (1000 ppm), 4171.25 kJ mol^–1^ (1500 ppm), 5229.18 kJ mol^–1^ (2000 ppm), and 6061.06 kJ mol^–1^ (2500 ppm). These values indicate a repulsion between the OXC molecules, which increases when the concentration of the OXC concentration increases. This repulsion is occasioned by the high positive electrostatic energy values in Table S2 (Supporting Information). These increases in positive values may be associated with the formation of OXC clusters observed in the 1500, 2000, and 2500 ppm systems in Figure 7c–e, respectively. On the other hand, these OXC clusters also stabilize the interactions between the OXC molecules due to a reduction of van der Waals energy values (Table S2 in the Supporting Information). Therefore, the IPE results concerning the interactions between the OXC molecules indicated an increase in the repulsion of these molecules, especially for high OXC concentrations.

**Figure 6:**
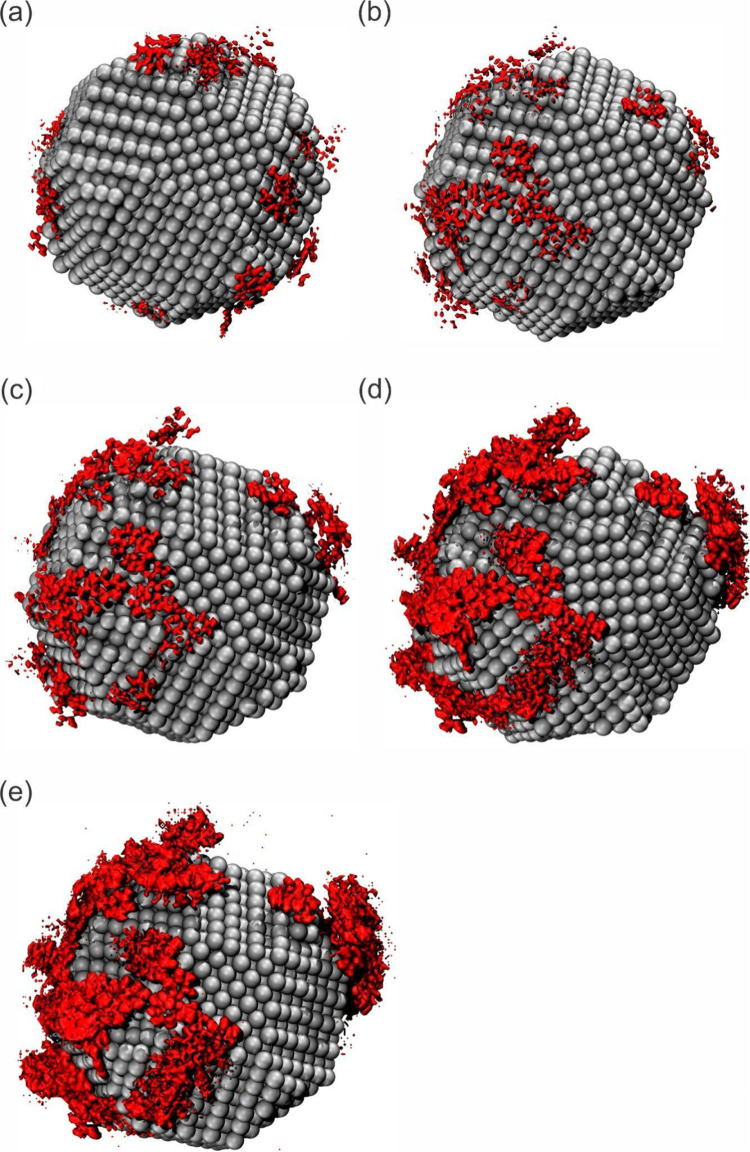
SDF in the last frame of simulation (100 ns) of the (a) 500 ppm, (b) 1000, (c) 1500, (d) 2000, and (e) 2500 ppm systems.

The spatial distribution function (SDF) analysis (Figure 6) was performed to analyze how the OXC molecules are coated around AgNP in the last frame (100 ns) of the simulation. In all of the systems (Figure 6a–e) analyzed, the OXC density is coated around the AgNP. Furthermore, analyzing the effect of the increased OXC concentration, it was observed that there was a greater density of the OXC around the AgNP. Therefore, the SDF results indicated the highest coated OXC molecules around the AgNP when the OXC concentration increased.

### 3.2 Weak Interactions Analysis Results

**Figure 7:**
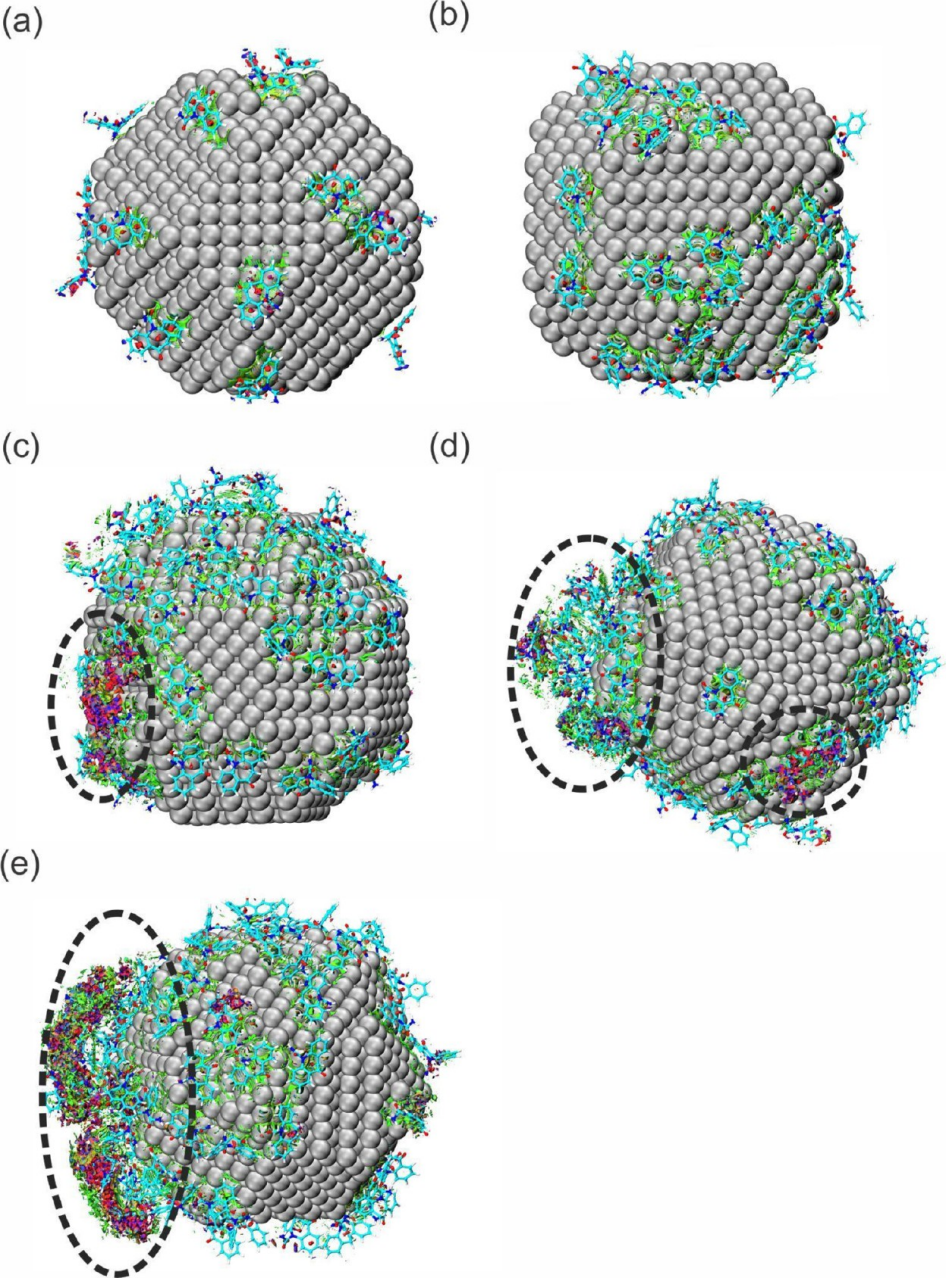
aNCI isosurface map of OXC-AgNP and OXC-OXC interactions of the (a) 500 ppm, (b) 1000 ppm, (c) 1500 ppm, (d) 2000 ppm, and (e) 2500 ppm systems.

The NCI results were based on the 50 ns dynamics result, with the AgNP coordinates frozen after the production stage to verify only the OXC-AgNP and the OXC-OXC interactions during the MD simulation process. Therefore, the sodium and chlorine ions and water were removed for aNCI analysis. The RDG isosurface of each system (500–2500 ppm) is calculated by averaging the dynamics frames. The strong attractive interactions, such as hydrogen bonds, are colored blue, medium force interactions, such as van der Waals forces, are colored green, and repulsive interactions are colored red. According to Figure 7, the aNCI results indicate that van der Waals forces mainly govern the direct interactions between the OXC and AgNP molecules. However, from a concentration of 1500 ppm (Figure 7c), there are significant interactions among the clusters of the OXC molecules. These interactions are also observed with increasing concentrations of 2000 and 2500 ppm (Figure 7d,e, respectively), and this is manifested by the coloration of the blue and red isosurface between the OXC-OXC interaction, caused by the attraction and repulsion occurring throughout the MD process (conform seen in the IPE results), in which the OXC-AgNP sites create an aggregated environment, facilitating an OXC-OXC interaction around the nanoparticle.

**Figure 8:**
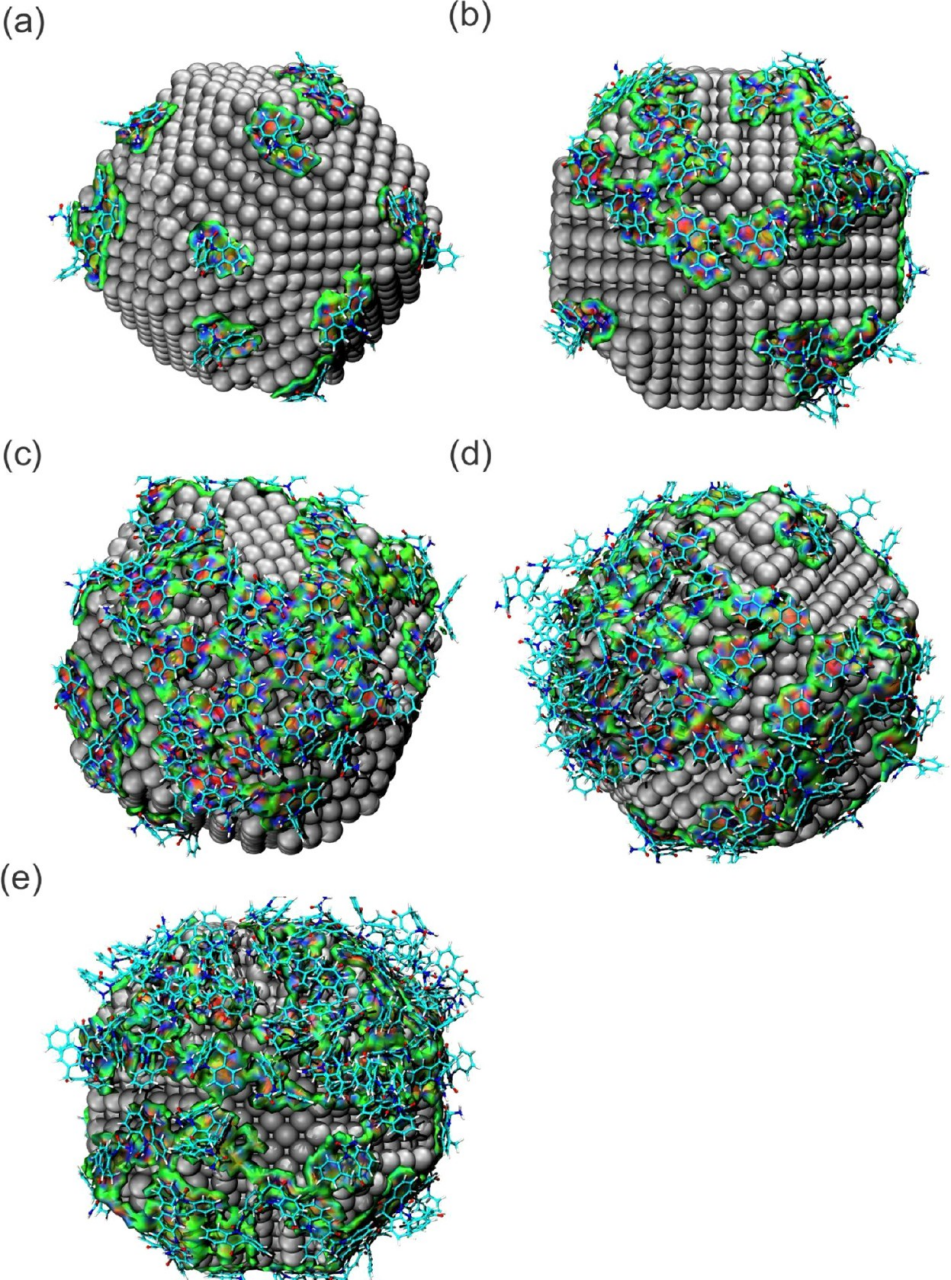
aIGM isosurface map of OXC-AgNP interactions of the (a) 500 ppm, (b) 1000 ppm, (c) 1500 ppm, (d) 2000 ppm, and (e) 2500 ppm systems.

The IGM method has been very relevant for analyzing weak interactions as complementary to the NCI method.^50,51,55−56,57,58^ Therefore, aIGM was used to analyze only the interactions between the OXC and AgNP and verify the nature of these interactions. Figure 8 shows that the isosurface corroborates the aNCI data, as there is a predominance of weak, noncovalent, van der Waals interactions between the OXC-AgNP, highlighted by the green color, and the area of these interactions increases from 500 to 1000 ppm and from 1000 to 1500 ppm. However, the OXC-AgNP interaction isosurface does not increase in the same proportion for the variation from 1500 to 2000 ppm and 2500 ppm, with even a greater proportional amount of red-colored surfaces being observed, indicating a greater repulsive interaction. Therefore, the interaction of the drug with the nanoparticle was significant up to a concentration of 2500 ppm. Still, the proportion of increase in these interactions is less effective from 1500 ppm onward.

**Figure 9:**
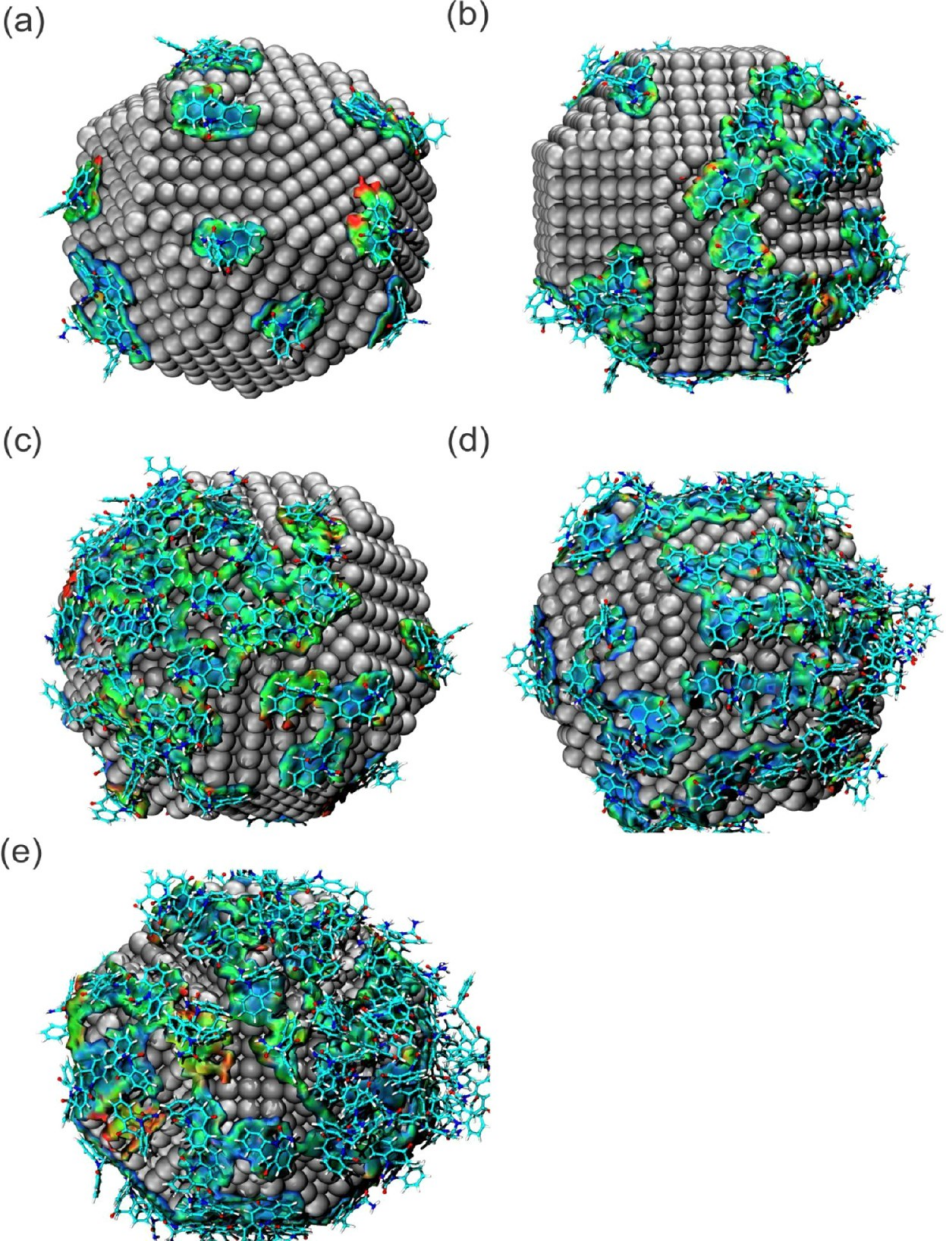
TFI isosurface map of OXC-AgNP interactions of the 500 ppm (a), 1000 ppm (b), 1500 ppm (c), 2000 ppm (d), and 2500 ppm (e).

The TFI indicates the stability of the interaction throughout the MD. The stability of the interactions of interest, OXC-AgNP, was analyzed using data obtained from aIGM. Thus, Figure 9 shows the regions of the interactions of the OXC-AgNP throughout the MD at all concentrations, indicating a high stability of the interaction, proving to be a very stable noncovalent interaction for the transport of the OXC.

## 4 Conclusions

The coating of icosahedral AgNP with five concentrations of OXC, a drug widely used in epilepsy treatment, was investigated using MD simulations and weak interactions analysis (aNCI, aIGM, and TFI). Our investigations through MD simulations showed that the OXC has a high interaction potential with the AgNP, especially for the 2500 ppm system. This interaction will occur mainly through the nitrogen atom (N_1_) of the OXC molecule, independent of the concentration. Furthermore, a high coating by the OXC molecules was observed around the AgNP. The weak interactions analysis indicated that the OXC molecules interact predominantly by van der Waals forces, corroborated by the IPE, aNCI, and aIGM data. At a concentration of 1500 ppm, a cluster of OXC is formed around the AgNP, visually observed in the aNCI. In addition, the TFI data confirms the stability of the OXC-AgNP interaction throughout the dynamics. Therefore, based on MD simulation investigations, we can suggest that these AgNPs can be used as a drug delivery vehicle to increase the efficacy of OXC by improving the controlled release of the drug, aiming for better efficacy in treating epilepsy.

## Supporting Information Available

Interaction potential energy between the AgNP and OXC molecules for the 500, 1000, 1500, 2000, and 2500 ppm systems (Table S1); and electrostatic, van der Waals, and interaction potential energies between the OXC molecules for the 500, 1000, 1500, 2000, and 2500 ppm systems (Table S2) (PDF)
