# Supplementary material for: Molecular Nanoinformatics Approach Assessing the Coating Oxcarbazepine (OXC) Drug on Silver Nanoparticles
Source: ACS Omega. 2024 Nov 5;9(46):46091–103. doi: 10.1021/acsomega.4c06366 (PMC11579947; doi:10.1021/acsomega.4c06366)
Supplement: Supplementary file 1 — ao4c06366_si_001.pdf [file ao4c06366_si_001.pdf]

Supporting Information

Molecular nanoinformatics approach assessing the coating oxcarbazepine (OXC) drug on silver nanoparticle

Norberto de Kássio Vieira Monteiro[1,*], Lucas Lima Bezerra[1], Leonardo P. da Silva[1], and Richele Machado[2]

[1]Federal University of Ceará, Department of Analytical Chemistry and Physical Chemistry, Science Center, 60020-181, Fortaleza, CE, Brazil.

[2] Christus University Center, 60160-230, Fortaleza, CE, Brazil.

*Corresponding author.
E-mail address: norbertokv@ufc.br.

Table S1

Interaction Potential Energy between the AgNP and OXC molecules for the 500, 1000, 1500, 2000, and 2500 ppm systems.

Systems	Interaction Potential Energy / kJ mol^{-1}			
	Replicate 1	Replicate 2	Replicate 3	Average
500 ppm	-3531.59 (±88.77)	-3222.26 (±98.78)	-3478.18 (±117.36)	-3410.68
1000 ppm	-6129.37 (±108.53)	-6017.25 (±85.60)	-6087.44 (±79.85)	-6078.02
1500 ppm	-8816.30 (±160.60)	-8956.47 (±115.42)	-8094.20 (±226.59)	-8622.32
2000 ppm	-10710.20 (±201.28)	-10212.20 (±206.24)	-10358.70 (±235.80)	-10427.03
2500 ppm	-10797.50 (±190.24)	-12717.80 (±181.15)	-12787.10 (±188.64)	-12100.80

Table S2 Electrostatic, van der Waals, and interaction potential energies between the OXC molecules for the 500, 1000, 1500, 2000, and 2500 ppm systems.

Systems	Replicate	Electrostatic Energy / kJ mol^{-1}	van der Waals Energy / kJ mol^{-1}	IPE / kJ mol^{-1}	Average IPE
	1	1863.69 (±22.45)	-162.41 (±67.78)	1701.28 (±92.27)	
500 ppm	2	1837.00 (±31.85)	-200.37 (±34.31)	1636.63 (±44.29)	1670.54
	3	1855.60 (±22.07)	-181.88 (±48.93)	1673.72 (±56.76)	
	1	3525.87 (±38.38)	-569.96 (±67.78)	2955.91 (±101.38)	
1000 ppm	2	3534.57 (±43.11)	-602.12 (±47.18)	2932.45 (±60.76)	2948.87
	3	3556.96 (±42.02)	-598.71 (±88.91)	2958.25 (±117.89)	
	1	5317.00 (±26.65)	-1184.75 (±44.58)	4132.25 (±154.52)	
1500 ppm	2	5411.24 (±44.94)	-1118.83 (±34.66)	4292.41 (±65.28)	4171.25
	3	5367.92 (±44.95)	-1278.82 (±117.60)	4089.10 (±145.60)	
	1	7138.57 (±54.93)	-1843.86 (±163.37)	5294.71 (±183.36)	
2000 ppm	2	7047.72 (±85.21)	-1958.68 (±171.32)	5089.04 (±212.07)	5229.18
	3	7158.64 (±58.49)	-1854.86 (±138.82)	5303.78 (±155.54)	
	1	8713.12 (±100.53)	-3003.89 (±213.85)	5709.23 (±288.67)	
2500 ppm	2	8863.76 (±76.55)	-2589.09 (±191.93)	6274.67 (±239.67)	6061.06

	3	8755.85<br>(±98.62)	-2556.57<br>(±206.03)	6199.29<br>(±243.56)
